# The impact of tele-expertise in oncology: current state and future perspectives

**DOI:** 10.3389/fdgth.2025.1552182

**Published:** 2025-08-29

**Authors:** Pierre Heudel, Mona Amini-Adle, Julien Anriot, Gualter Vaz, Claire Laine, Aline Desoutter, Hugo Crochet

**Affiliations:** ^1^ Department of Medical Oncology, Centre Léon Bérard, Lyon, France; ^2^ Department of Surgical Oncology, Centre Léon Bérard, Lyon, France; ^3^ Department of Information Systems and Data Management, Centre Léon Bérard, Lyon, France

**Keywords:** tele-expertise, telemedicine, cancer, digital health, teledermatology, telepathology

## Abstract

**Background:**

Tele-expertise, the asynchronous exchange of medical expertise via secured digital platforms, is revolutionizing healthcare delivery. By overcoming geographical and logistical barriers, it enables timely access to specialized care and supports multidisciplinary decision-making, particularly in oncology. Its adoption has accelerated with the COVID-19 pandemic and regulatory changes, improving patient outcomes by facilitating efficient diagnosis and treatment.

**Materials and methods:**

An anaysis of peer-reviewed studies published in the past decade was conducted using PubMed, MEDLINE, and the Cochrane Library. The inclusion criteria focused on studies evaluating tele-expertise for remote consultations, imaging interpretation, pathology evaluation, and multidisciplinary tumor boards. Methodological quality, including study design, sample size, and reliability of outcomes, was assessed. Cross-referencing and manual searches were performed to ensure comprehensive coverage.

**Results:**

The review demonstrated that tele-expertise improves access to specialized consultations, enhances diagnostic accuracy, and expedites clinical decision-making. Applications in oncology include remote imaging interpretation and support for multidisciplinary teams. Challenges identified include the transmission of large imaging files, the need for secure and robust IT infrastructure, and training healthcare providers. Ethical considerations, such as data privacy and medical liability, remain key barriers.

**Conclusion:**

Tele-expertise is transforming healthcare by enabling equitable access to specialized care and fostering collaboration in oncology. Addressing challenges related to infrastructure, training, and ethical issues is critical to maximizing its potential. The integration of AI and further advancements in telemedicine platforms will enhance its role in delivering high-quality, timely care globally.

## Introduction

In today's dynamic healthcare landscape, telemedicine is transforming the delivery of medical services across various specialties, including oncology, dermatology, dentistry, and pathology (1). This digital evolution is dismantling traditional geographical and logistical barriers, enabling enhanced access to specialized care worldwide. Central to this transformation is tele-expertise, defined as the asynchronous exchange of medical opinions and expertise between healthcare professionals via digital technologies (2, 3). This allows general practitioners and non-specialist oncologists to consult remotely with specialists, improving decision-making and patient care. Accelerated by the COVID-19 pandemic and regulatory adaptations, tele-expertise has gained significant traction, particularly in Europe and the United States (4–6). By leveraging digital platforms, telemedicine not only expedites diagnosis and treatment but also improves patient outcomes by ensuring timely interventions (7). From teledermatology and teledentistry to telepathology and teleoncology, these innovative applications extend specialist services to underserved areas, streamline workflows, reduce costs, and minimize risks such as infectious disease transmission. This study provides a comprehensive analysis of the transformative role of tele-expertise in oncology by systematically reviewing recent evidence on its implementation across clinical settings, evaluating its contributions to improved access, diagnostic accuracy, and multidisciplinary collaboration, identifying persistent barriers such as technical infrastructure and ethical concerns, and outlining future directions including AI integration and policy development to enhance its integration into mainstream cancer care.

## Materials and methods

To evaluate the role and impact of tele-expertise in cancer care, we conducted a comprehensive literature review focused specifically on oncology-related applications of tele-expertise. The search was conducted using databases such as PubMed, MEDLINE, and the Cochrane Library, with the query “tele expertise AND cancer”, limited to articles published between January 2014 and May 2025. Filters were applied to include studies written in English or French, involving adult cancer patients and/or healthcare professionals working in oncology. Eligible article types encompassed clinical studies, reviews, scoping reviews, implementation studies, and case series. The initial search returned 50 articles. After sorting results by publication date, we retained only those explicitly referencing the use of tele-expertise in oncology settings. Core applications examined included remote second opinions, virtual multidisciplinary tumor boards (MTBs), collaborative imaging or pathology interpretation, and coordination of care across institutions.

**Figure 1:**
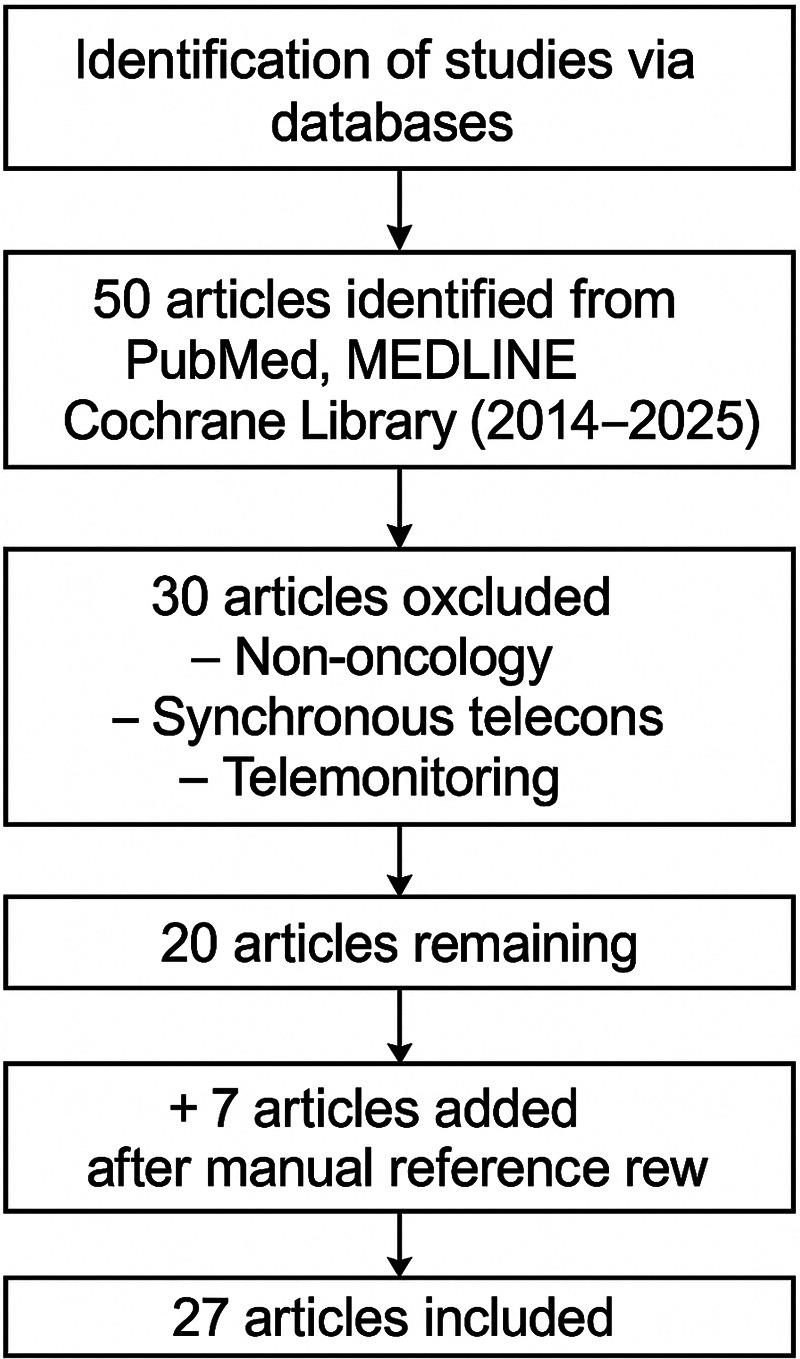
PRISMA flow diagram.

To enhance the comprehensiveness of our analysis, we manually screened reference lists and bibliographies of the selected studies to identify additional relevant articles not retrieved in the initial search. Each study was evaluated according to its design, sample size, population characteristics, and relevance to clinical endpoints such as diagnostic accuracy, access to expert input, time to treatment, and stakeholder satisfaction. Methodological quality was assessed using principles derived from standard evidence synthesis frameworks, such as PRISMA (Figure 1).

Importantly, we observed that the literature specifically addressing tele-expertise in oncology—distinct from broader themes of teleconsultation and telemonitoring—is relatively limited. Many publications conflate synchronous teleconsultations and remote surveillance platforms with asynchronous expert input, which narrows the field of evidence truly dedicated to tele-expertise as defined in this review. This relative scarcity of targeted research underscores the need for clearer conceptual boundaries and more dedicated studies examining the operational, clinical, and ethical dimensions of tele-expertise in cancer care.

## Results

The selected studies were grouped and analyzed by clinical domain to identify common patterns, benefits, and limitations of tele-expertise in oncology-related practice. A total of 27 articles were included in the qualitative synthesis, covering four main areas: dermatology, dentistry, pathology, and oncology. In teledermatology, studies consistently reported improved access to specialist advice, reduction in consultation delays, and high diagnostic concordance between remote and in-person assessments. The use of smartphone imaging and nurse-led protocols showed particular promise in underserved regions. In teledentistry, tele-expertise enabled earlier detection of oral lesions, particularly oral cancers, through mobile imaging and web-based diagnostic platforms. It also facilitated supportive care for oncology patients undergoing radiotherapy or chemotherapy. In telepathology, integration with digital slide imaging and artificial intelligence showed significant gains in diagnostic throughput and accuracy. Collaborative platforms allowed remote assessment and quality control, particularly in histopathology. In teleoncology, the implementation of remote tumor boards, inter-institutional consultations, and radiotherapy planning in low-resource settings contributed to improved care coordination, shorter time to treatment, and enhanced equity in cancer care delivery. Despite the diversity of approaches, several cross-cutting challenges were identified, including variability in technological infrastructure, medico-legal constraints, and the need for structured training and guidelines.

### Teledermatology

Teledermatology exemplifies the transformative potential of telemedicine in improving access to specialized care, particularly for underserved populations. It addresses the financial recognition of pre-existing activities, such as informal consultations that have always been part of clinical practice. Early research by Bouton et al. demonstrated how transmitting smartphone photographs from general practitioners to dermatologists significantly reduced waiting times for consultations, potentially enhancing early melanoma detection rates (8). This pioneering randomized trial highlighted the practicality of using simple digital tools to expedite patient triage and improve the management of skin cancer. This highlights how teledermatology can streamline care pathways, offering timely interventions and reducing unnecessary referrals through targeted triage. Building on this foundation, Chuchu et al. conducted a Cochrane review that confirmed teledermatology's effectiveness as a triage tool (9). The review demonstrated its ability to decrease the need for in-person visits while preserving diagnostic accuracy, making it a powerful strategy for reducing healthcare disparities and broadening the reach of specialist expertise to remote areas. Complementing these findings, Robin et al. investigated the application of tele-expertise in chronic wound management by dermatology nurses with advanced training (10). Their study emphasized the role of teledermatology in enabling timely interventions and ongoing monitoring for complex conditions, ultimately enhancing patient care. In addition to individual studies, comprehensive reviews have further validated teledermatology's reliability. Bourkas et al., through a systematic review and meta-analysis, found a high concordance between remote and in-person consultations, affirming teledermatology's diagnostic reliability, particularly in regions with limited access to specialists (11). Chow et al. synthesized evidence from systematic reviews to map the widespread influence of teledermatology on dermatological care (12). Their findings provided a structured perspective on its effectiveness and identified areas for further exploration, suggesting a robust framework for integrating teledermatology into routine clinical practice. Despite these successes, challenges remain. van Sinderen et al. highlighted variability in study designs and outcomes in teledermoscopy research, calling for standardized methodologies to enhance reliability and comparability (13). However, their long-term retrospective analysis of over 18,000 teledermoscopy consultations in the Netherlands underscored the enduring value of teledermatology as a dependable component of telehealth. Teledermatology has proven particularly impactful in underserved regions. Dietrich et al. demonstrated how tele-expertise significantly improved dermatologic care in low-physician-density areas, offering essential support to general practitioners (14). Finally, Senet emphasized the importance of integrating teledermatology training into general practitioner education to bridge the gap between primary care and specialized dermatology (15). This approach aims to enable earlier interventions and better management of skin conditions through enhanced diagnostic skills. Collectively, these studies underscore the transformative potential of teledermatology in advancing healthcare delivery and improving patient outcomes. By enhancing access, streamlining workflows, and ensuring timely interventions, teledermatology has become an essential tool in modern dermatological practice (Table 1).

**Table 1:** Teledermatology advantages and inconvenients.

Advantages	Details (advantages)	Limitations	Details (limitations)
Improved access to care	Extends dermatology services to underserved or remote regions.	Dependence on technology	Requires stable internet access and high-quality digital imaging.
Reduced wait times	Expedites specialist consultations and accelerates patient triage.	Lack of physical examination	Tactile information (texture, relief) is not accessible remotely.
Cost efficiency	Reduces travel costs and resource use for both patients and institutions.	Privacy and data security	Raises concerns over patient data confidentiality and transmission safety.
Continuity of care	Enables follow-up assessments without requiring in-person visits.	Variable diagnostic accuracy	Diagnostic reliability may vary based on image quality and provider skills.
Educational value	Supports training and upskilling of general practitioners remotely.	Limited patient interaction	Reduces opportunities for direct rapport and communication.

### Teledentistry

Teledentistry is revolutionizing the field of dental medicine by leveraging digital technologies to enhance the detection and management of oral diseases, particularly oral cancers. Studies such as those conducted by Vigarios et al. (16) and Niknam et al. (17) provide compelling evidence of the benefits of integrating tele-expertise and web-based diagnostic tools into dental practice. Vigarios et al. discuss how tele-expertise in dentistry can expedite the early detection of oral malignancies, in a field without an oral cancer screening campaign and with few health professionals trained in early detection. This could potentially leading to more timely and effective interventions. They also propose that such technologies could reduce the average time to diagnosis, thus improving patient prognosis through earlier treatment. In a practical application of mobile technology, Haron et al. explored the use of mobile phone imaging for early detection of oral cancer in low-resource settings (18). Their study demonstrated that images captured by non-specialists using standard mobile phones could achieve a diagnostic concordance rate of over 70% when compared to clinical oral examinations conducted by professionals. This suggests that mobile phone imaging can be a viable screening tool in areas where access to specialized healthcare is limited. Further emphasizing the utility of digital tools, Niknam et al. assessed the usability and reliability of a web-based teledentistry tool for diagnosing oral lesions (17). Their findings indicated a high user satisfaction rate, with over 80% of participants finding the tool easy to use. Additionally, the diagnostic accuracy of the tool was comparable to in-person assessments, with a reliability coefficient of 0.85, underscoring the potential of web-based platforms to support remote dental diagnostics effectively. Another comprehensive review by Niknam et al. focused on the technological aspects and implementation recommendations for teledentistry (19). They outlined critical factors necessary for successful integration, including robust internet connectivity, high-quality imaging devices, and secure data transmission protocols to protect patient privacy. Collectively, these studies not only demonstrate teledentistry's potential to improve oral health outcomes but also highlight the practical considerations and technological requirements for its successful adoption. In addition to the early diagnosis of oral cancer, teledentistry in oncology helps the attending dentist to support the oral health care of patients during and after oncological therapies. Some treatments have side effects requiring specific dental protocols. Teledentistry makes this expertise affordable outside of reference centers. By providing accessible, efficient, and comprehensive care, teledentistry stands to significantly impact dental medicine, particularly in underserved and remote communities worldwide, aligning with broader global health initiatives aimed at reducing healthcare disparities.

### Telepathology

The integration of telepathology and digital pathology with artificial intelligence (AI) represents a transformative leap in medical diagnostics, significantly enhancing the accuracy and efficiency of pathological assessments. A recent study by Battazza et al. discusses the inevitable future of combining these technologies (20). Their research highlights how AI can automate image analysis, reduce diagnostic errors, and facilitate faster decision-making processes. The paper projects an increase in diagnostic accuracy by up to 20% when AI is integrated into digital pathology workflows, thus enabling more precise and rapid disease identification. Jain et al. provide an extensive overview of current and emerging applications of Whole Slide Imaging (WSI) technology in pathology (21). WSI allows for high-resolution imaging of entire pathology slides, which is essential for detailed analysis. Their research emphasizes the potential of WSI to revolutionize pathological assessments by enabling pathologists to examine slides digitally, thus reducing the need for physical slide handling and enabling remote consultations. The study notes that WSI has been instrumental in increasing the throughput of pathological assessments by approximately 30%, significantly enhancing the productivity of pathology departments. Further advancing the field, Mosquera-Zamudio et al. detail the protocol for the GLORIA program in Colombia, which aims to globalize a telepathology network enhanced by AI (22). This initiative seeks to bridge the gap between remote areas and advanced diagnostic facilities. The GLORIA program is designed to connect various institutions and provide them with access to AI-powered diagnostic tools, facilitating a more inclusive approach to pathology services. The program anticipates a reduction in diagnostic turnaround times by as much as 40%, demonstrating the substantial impact of integrating AI into telepathology networks. These studies illustrate the robust potential of AI in transforming pathology services, providing a glimpse into a future where digital and AI-enhanced pathology systems could become standard practice across the globe. This shift not only promises to improve diagnostic accuracy and efficiency but also aims to democratize access to quality healthcare diagnostics, thereby reshaping the landscape of medical pathology.

### Teleoncology

The expansion of teleoncology within the Veterans Health Administration (VHA) represents a pivotal advancement in delivering cancer care to U.S. veterans, particularly those residing in remote or underserved areas. Parikh et al. (23) and Zullig et al. (24) have explored various models of care within the VHA's teleoncology services, shedding light on how these innovations enhance patient experiences and access to care. Parikh et al. detail the diverse teleoncology care models being implemented across the VHA, focusing on their efficacy in providing timely and comprehensive oncology consultations (23). Their findings suggest that these models significantly improve the continuity of care and patient satisfaction, as they reduce travel burdens and provide more immediate access to oncology specialists. Zullig et al. further emphasize the scalability of the VHA's National TeleOncology Service, highlighting its role in standardizing cancer care delivery across various healthcare facilities (24). This service facilitates real-time oncology consultations and follow-ups via secure video conferencing tools, which not only streamlines the care process but also ensures that veterans receive consistent and high-quality oncological advice regardless of their geographical location. Their study reports a 25% increase in patient engagement and a 15% improvement in treatment adherence among participants utilizing teleoncology services. Additionally, Salem et al. discuss the broader implications of telemedicine in global health contexts, specifically in expanding access to radiotherapy for cancer patients in low-resource settings (25). Their research advocates for the integration of telemedicine into radiotherapy planning and follow-up, which could potentially bridge the gap in radiotherapy access worldwide. They propose that telemedicine could expedite the planning process and enhance the quality of care through remote expert collaboration, ultimately increasing the global radiotherapy coverage by up to 30%. These studies collectively illustrate the transformative potential of teleoncology and telemedicine in enhancing cancer care delivery. They highlight the importance of technology in overcoming geographical barriers, improving patient outcomes, and equalizing access to essential healthcare services across diverse populations and settings.

## Discussion

This study reviewed twenty-seven articles across four major clinical domains: teledermatology, teledentistry, telepathology, and teleoncology. In teledermatology, access to specialists improved, consultation delays decreased, and diagnostic concordance remained high. In teledentistry, mobile imaging and web-based platforms enabled earlier detection of oral lesions, including cancers. In telepathology, digital slide imaging and AI integration significantly increased diagnostic throughput and accuracy in histopathology. In teleoncology, remote tumor boards and inter-institutional consultations improved care coordination and reduced time to treatment, despite ongoing technical, legal, and training challenges.

### Interpretation of findings relative to objectives

The main objective was to assess how telemedicine modalities have influenced diagnostic performance, workflow efficiency, and patient outcomes in oncology-related fields. Across all four domains, telemedicine technologies demonstrated both time-saving benefits and maintained—or even improved—diagnostic accuracy compared to traditional methods. For teledermatology and teledentistry, earlier intervention potential emerges from reduced referral delays. In telepathology, digital workflows and AI assistance address histopathology bottlenecks. In teleoncology, virtual collaboration directly streamlines decision-making for complex cancer cases.

### Limitations and challenges of tele-expertise

Tele-expertise has shown potential to enhance access to specialized care remotely in certain contexts, but several challenges and limitations must be addressed to maximize its impact. One key concern is the quality of transmitted data, which directly affects the accuracy of diagnoses and treatment plans. For instance, in dermatology, static photographs are often sufficient, but the use of dermoscopy by requesting physicians could greatly enhance diagnostic precision and improve skin cancer detection, optimizing teledermatology's value (13). In contrast, oncology relies heavily on volumetric imaging modalities such as CT scans, MRIs, and PET scans, which involve large file sizes and complex formats. The transmission of these datasets often exceeds the capabilities of available infrastructure, particularly in resource-constrained settings, where slow transfer rates and incompatible formats remain frequent obstacles. One practical limitation is the uneven training and digital literacy among referring physicians, which can affect the quality and relevance of tele-expertise requests. General practitioners and other non-specialists need to be equipped with the skills to recognize skin tumors and prioritize relevant cases, thereby reducing the saturation of tele-experts with non-urgent or irrelevant requests. Similarly, tele-experts must adapt their workflows to handle the increasing demand. This includes dedicating specific time slots for teleconsultations and developing strategies to manage urgent cases efficiently, preventing the system from becoming overwhelmed. In oncology, the inherently multidisciplinary nature of decision-making presents additional challenges. Effective tele-expertise requires coordination among various specialists, yet scheduling, platform interoperability, and workload constraints often limit real-time collaboration. This necessitates well-coordinated telemedicine platforms capable of supporting real-time, high-quality discussions and simultaneous access to imaging and pathology reports (26). Beyond these technical and workflow considerations, the robustness of IT infrastructure and user training remain pivotal. In practice, many platforms lack sufficient integration or stability, making data sharing and security compliance challenging for institutions with limited IT support. Finally, ethical and legal challenges—including issues of medical responsibility, liability in virtual consultations, and data privacy—remain significant barriers to broader adoption. Addressing these technical, operational, and ethical limitations is essential to fully realize the potential of tele-expertise, ensuring equitable access to specialized care and improving healthcare delivery across various specialties.

### Practical recommendations for implementation

To address persistent barriers in the deployment of tele-expertise, we propose a set of actionable recommendations structured around four critical domains: training, file transmission, data privacy, and access to specialists. First, in terms of training, structured continuing education should be developed to familiarize both general practitioners and specialists with tele-expertise tools and workflows (27). This could include simulation-based learning environments and the integration of tele-expertise modules into medical curricula and continuing professional development programs. Second, file transmission of large and complex datasets—particularly in oncology, where imaging formats such as CT, MRI, and PET are prevalent—requires optimized infrastructures. We advocate for the use of advanced compression algorithms (e.g., JPEG2000), DICOM-compatible cloud storage, and secure streaming platforms to ensure real-time accessibility without compromising diagnostic quality (28, 29). Third, regarding data privacy, platforms must adhere to GDPR-compliant standards, including the implementation of end-to-end encryption, granular access control mechanisms, and automated data anonymization processes (30). These safeguards are essential to maintain patient trust and uphold legal obligations. Finally, to enhance access to specialists, we suggest the establishment of rotating tele-expert panels and regionally coordinated scheduling systems (31). AI-assisted triage algorithms could further support the prioritization of cases, ensuring efficient allocation of expert resources. Collectively, these strategies aim to support the sustainable and ethical scaling of tele-expertise in oncology and beyond.

### Future perspectives

Looking ahead, the future of tele-expertise is promising, with several developments on the horizon that could further its impact. The integration of Artificial Intelligence (AI) stands out as a particularly transformative advancement. AI can augment tele-expertise platforms by providing predictive analyses and sophisticated data-based recommendations, thereby enhancing decision-making processes and diagnostic accuracy (32). Additionally, the expansion of multidisciplinary tele-expertise, involving radiologists, pathologists, and dermatologistes, promises a more integrated and comprehensive approach to patient care, facilitating a seamless collaboration across different specialties. To support these advancements, strengthening training and collaborative practices will be essential. Continuous education programs that include practical training on tele-expertise tools will be crucial for familiarizing practitioners with these technologies and encouraging their adoption, ensuring that tele-expertise remains at the forefront of improving healthcare delivery.

## Conclusion

While tele-expertise holds significant promise for improving access to specialized care and supporting clinical decision-making, its transformative impact on healthcare systems remains uneven and context-dependent. Although some settings have reported reduced delays and enhanced coordination, these benefits are not universally observed and often depend on robust technical infrastructure, clear procedural workflows, and active stakeholder engagement. Persistent challenges—including data quality, interoperability, medico-legal uncertainties, and disparities in digital literacy—continue to limit widespread and equitable implementation. Furthermore, the lack of standardization in practices and outcome reporting hinders broader generalization of results. Addressing these barriers through targeted investment, regulatory clarity, and workforce training is essential to gradually unlock the potential of tele-expertise in oncology. However, its integration into routine care should be guided by evidence-based approaches, continuous evaluation, and realistic expectations tailored to local constraints.

## Data Availability

The raw data supporting the conclusions of this article will be made available by the authors, without undue reservation.
